# Foreign language anxiety and dependency distance in English–Chinese interpretation classrooms

**DOI:** 10.3389/fpsyg.2022.952664

**Published:** 2022-10-26

**Authors:** Jackie Xiu Yan, Junying Liang

**Affiliations:** ^1^Department of Linguistics and Translation, City University of Hong Kong, Kowloon, Hong Kong SAR, China; ^2^Department of Linguistics, Zhejiang University, Hangzhou, China

**Keywords:** foreign language anxiety, cognitive load, dependency distance, interpretation, language learning, interpretation learning, sight translation, consecutive interpretation

## Abstract

Foreign language anxiety (FLA) has been identified as a crucial affective factor in language learning. Similar to the situation in language classes, university students in interpretation classes are required to perform in a foreign language when their language skills are inadequate. Investigations are needed to determine the specific impact of FLA on interpretation learning. This study investigated the effects of the specific interpretation classroom FLA (ICFLA) on interpretation learning and dependency distance (DD) as an indicator of learners’ cognitive load. The participants were 49 undergraduate and graduate students enrolled in English–Chinese interpretation classes at a university in Hong Kong. The results showed a significant negative correlation between ICFLA levels and consecutive interpretation achievement scores. ICFLA was also negatively correlated with DD in consecutive interpretations. Four factors underlying ICFLA were identified. The findings of this study would provide useful insights for researchers and educators to understand the nature and effect of FLA in different settings.

## Introduction

Foreign language anxiety (FLA) has been identified as a crucial affective factor in students’ language learning (Fallah, 2017; Abdurahman and Rizqi, 2020). College students’ foreign/second language skills are still developing. When they are required to communicate in this language, they tend to feel anxious. Even though their command of the language is immature, their “individual communication attempts will be evaluated according to uncertain or even unknown linguistic and sociocultural standards, second language communication entails risk-taking and is necessarily problematic” (Horwitz et al., 1986, p. 31). Anxiety has been documented in both foreign language and interpretation classes (Chiang, 2010), and the negative effect of FLA on language learning has been found by many studies (e.g., Cheng et al., 1999; Yan and Wang, 2001; Argaman and Abu-Rabia, 2002; Yan and Detaramani, 2008). Only a few studies have been conducted on the effect of FLA on the learning of interpretation see Chiang (2009); Chiang (2010); Wei et al. (2020). More studies are needed to investigate FLA in interpretation classes. More importantly, as both language and interpretation learning involve complex cognitive operations, it is important to see the role of FLA in affecting cognitive functions. This study is an attempt in this direction: It investigated the effects of FLA on interpretation learning and cognitive load. The findings of this study are expected to advance our knowledge of FLA in interpretation and language learning.

### Literature review

Foreign language anxiety (FLA) is defined as a “distinct complex of self-perceptions, beliefs, feelings, and behaviors related to classroom language learning arising from the uniqueness of the language learning process” (Horwitz et al., 1986, p. 128). According to this definition, FLA is a situation-specific construct (MacIntyre, 1999; Teimouri et al., 2019), which suggests that people who do not normally feel anxious may be struck by anxiety in language classrooms. The Foreign Language Classroom Anxiety Scale (FLCAS), constructed by Horwitz et al. (1986) based on this understanding, has been used as a standard instrument in studies of FLA (Horwitz, 2010). With the breakthrough in conceptualizing this construct and the aid of this highly valid and reliable instrument, researchers have been able to conduct systematic investigations (e.g., MacIntyre and Gardner, 1991; Phillips, 1992; Aida, 1994; Saito and Samimy, 1996; Cheng et al., 1999; Yan and Detaramani, 2008; Liu and Li, 2019), and a consistent negative correlation has been found between FLA and achievement. The adverse effect of FLA on language achievement has been confirmed by studies on the learning of different languages (e.g., Kitano, 2001; Yan and Wang, 2001; Kondo and Yang, 2004; Matsuda and Gobel, 2004; Elkhafaifi, 2005; Frantzen and Magnan, 2005), and various aspects of language proficiencies (e.g., Oh, 1992; Cheng et al., 1999; Saito et al., 1999; Kim, 2000; Sellers, 2000; Argaman and Abu-Rabia, 2002; Elkhafaifi, 2005; Liu, 2006).

Based on the understanding of the negative influence of FLA on language performance, some researchers have devoted themselves to the search for sources of FLA (e.g., Young, 1991; Liu, 2006; Yan and Horwitz, 2008). Additionally, more and more studies have focused on the relationship between language anxiety and other learning variables (Oteir and Al-Otaibi, 2019). For example, studies on the relationship of FLA with the following variables: Willingness to communicate (Liu and Jackson, 2008; Rastegar and Karami, 2015; Yan et al., 2018; Kalsoom et al., 2020; Zhou et al., 2020), learning style (Bailey et al., 1999), self-efficacy (Mills et al., 2006; Eginli and Solhi, 2020; Wang et al., 2022), self-confidence (Bensalem and Thompson, 2022), self-esteem (Rubio-Alcalá, 2017), learning autonomy (Ahmadi and Izadpanah, 2019), learning strategies (Abdurahman and Rizqi, 2020; Demir and Zaimoğlu, 2021), motivation (Duvernay, 2009; Saito et al., 2018; Alamer and Almulhim, 2021; Ismail and Hastings, 2021), learner beliefs (Aslan and Thompson, 2021), and personality (Dewaele, 2017; Šafranj, 2018; Šafranj and Zivlak, 2019).

When students feel anxious about learning or using a foreign language, their “worry and negative emotional reaction [are] aroused” (MacIntyre, 1999, p. 27), but it remains to be established why FLA exerts a debilitating influence on students’ language learning and performance. Researchers have proposed a number of mechanisms to explain the connection between the two. Krashen’s (1987) affective filter hypothesis suggests that when the affective filter is active, input information can be filtered out and fail to reach the learners’ brains. FLA may therefore activate and raise students’ affective filter and block their understanding of the input information. Researchers have observed and attempted to explain the interference of FLA with learners’ cognitive systems in each of their input, processing, and output stages see MacIntyre (1995); Shao et al. (2013). During the input stage, students’ attention might be attracted by task-irrelevant concerns, for example, fear of negative evaluation from their peers or teachers. When students cannot concentrate on the language learning task, the input information cannot reach their brains. Following the input stage, students’ speed and accuracy in storing information in the processing stage and the quality of their products in the output stage can also be affected by FLA (Abdurahman and Rizqi, 2020). MacIntyre and Gardner (1994) examined the effect of induced anxiety in the three stages of vocabulary learning. Stage-specific anxiety scales and stage-specific tasks were used to assess the “more specific, subtle effects of language anxiety” (p. 284). Their results showed that the effects of anxiety were evident during the input and processing stages, but not at the output stage of language learning. The increased effort during the previous stages ultimately reduced the effects of anxiety at the output stage. This supported Eysenck’s (1979) suggestion that increased effort may sometimes compensate for the effects of anxiety on the quality of observed performance.

Similar to the situation in language classes, university students in interpretation classes are required to perform in a foreign language even though their language skills are still inadequate (Yan et al., 2010). The use of interpretation classes to complement language learning and vice versa has attracted research interest. Interpretation has been used as a tool for foreign language teaching. It is not uncommon for students to sign up for interpretation training to improve their foreign language proficiency (Chiang, 2006), and interpretation classes are often part of language programs (Pan and Yan, 2012). Yagi’s (2000) research findings showed that simultaneous interpretation (SI) can be used as an effective tool for English as a foreign language (EFL) and confirmed that SI not only greatly contributes to students’ oral English fluency but is also effective in identifying their grammar and vocabulary abilities. Although Zannirato (2008) pointed out that language skills are not synonymous with translation skills, he investigated the feasibility of using various interpretation techniques in foreign language acquisition. It is therefore understood that foreign language training and interpretation training could be mutually beneficial. The relationship between foreign language teaching and interpretation training is interesting and interlocking.

Given the above similarity in foreign language learning, the impact of FLA on interpretation learning is worth exploring. There are many causes and various types of anxiety in interpretation classes, but only a few studies have been conducted on the impact of FLA on interpretation learning. Chiang; Chiang’s (2009; 2010) studies confirmed the negative correlation between FLA and interpretation learning achievement. These studies used the 33-item FLCAS developed by Horwitz et al. (1986), which is designed to measure students’ anxiety in foreign language classrooms. Most of the items on the scale are not suitable for interpretation classroom learning; thus, it is necessary to adapt the wording of these items and choose those that are relevant to interpretation classes.

Interpretation is a highly complex cognitive activity, and it has a close association with working memory (Liang et al., 2017). It is still not clear how anxiety in general and FLA, in particular, interfere with the interpretation process and affect interpreters’ performance.

Interpretation requires several patterns of attention-sharing and can overload the working memory, which tends to overwhelm students during interpreting practice. This cognitive overload could be a crucial factor mediating between FLA and interpreter performance. However, due to the absence of pertinent physiological approaches, it has been quite elusive to attempt to directly measure the working memory load or burden in such a complicated language processing activity. The dependency grammar approach (Hudson, 1995; Liu et al., 2017) comes right to the methodological rescue. Dependency grammar defines any grammatical relation in terms of a binary and asymmetric dependency relation between two syntactically related words, i.e., the head and the dependent, and accordingly proposes Dependency Distance (DD) as a measure of syntactic processing complexity. DD, coined by Heringer et al. (1980) and extended by Hudson (1995), is conceived simply as the linear word order difference between the head and dependent of a dependency. It has been theoretically and empirically validated as an effective means of quantifying the memory burden imposed on language processing that reflects the dynamic cognitive load of language processing demands (Hudson, 1995; Liu, 2008; Futrell et al., 2015; Liu et al., 2017; Wang and Liu, 2019; Jiang and Jiang, 2020).

As the interpreting task tends to push interpreters close to the saturation of their working memory capacity (Gile, 2009), it is plausible to assume stronger correlations between interpreters’ cognitive load and the DD of their interpretation, making DD a promising index to help investigate and quantify potential relationships between FLA and interpreting performance/learning. Because there is a universal tendency to reduce cognitive load, given the principle of least effort (Zipf, 1949), there is a tendency to syntactically restructure sentences to minimize the overall DD (Liu et al., 2017). According to Liang et al. (2017), this least effort tendency is found across different languages (Liu, 2008; Futrell et al., 2015), genres (Wang and Liu, 2017), and code-switching discourses (Wang and Liu, 2013), suggesting that it is affected by external constraints, especially that of limited working memory. Thus, this propensity can also affect interpretation processes. Their study found that different interpreting types “yield different DD” (Liang et al., 2017, p 1), and consecutive interpreting (CI) texts entail smaller DD than those of SI and read-out translated speech, indicating that the cognitive demands are higher for CI than that of SI and read-out translated speech.

**FIGURE 1:**
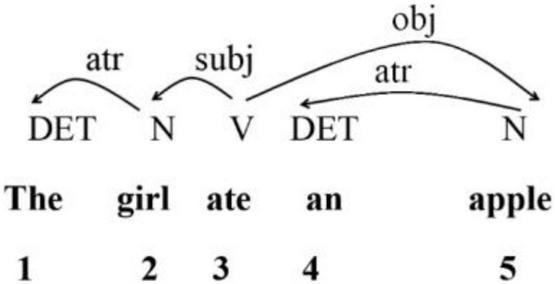
Dependency structure of sample sentence “*The girl ate an apple”* (Liang et al., 2017).

Based on Liang et al. (2017), we used in the present study a directed acyclic graph to present the dependency structure of a sentence as in Figure 1. The dependency analysis for the sentence “*The girl ate an apple”* is illustrated below.

Figure 1 shows the dependency relations between words in a sentence. For each pair of words linked by a dependency relation, one is called the dependent and the other the governor. The labeled arc extends from the governor to the dependent (Liu, 2008). The directed edge from governor to dependent illustrates the asymmetrical relation between these two units. The numbers below indicate the linear position of each word within the entire sentence. Liu et al. (2009) used the term Dependency Distance, and calculated the mean dependency distance (MDD) of a sentence with the following formula, where *n* is the number of words in a sentence and DDi is the dependency distance of the *i*-th syntactic link in the sentence:


(1) $$\text{MDD(the sentence)}\;\text{=}\frac{1}{\text{n}-1}\sum_{\text{i}=1}^{\text{n}-1}\left|{\text{DD}}_{\text{i}}\right|$$

This formula can also be used to examine the MDD of a text or a treebank:


(2) $$\text{MDD(the sample)}\;\text{=}\frac{1}{\text{n}-\text{s}}\sum_{\text{i}=1}^{\text{n}-\text{s}}\left|{\text{DD}}_{\text{i}}\right|$$

where *n* is the total number of words in the sample, *s* is the total number of sentences in the sample, and DDi is the dependency distance of the *i*-th syntactic link of the whole text.

Thus, in the sample sentence, *The girl ate an apple*, a series of DDs can be obtained: 1 1 0 1 2. Each DD is obtained by subtracting the number of the word and that of its governor. Then, using Formula (1), the MDD of this sentence is obtained as 5/4 = 1.25.

This study investigated the effects of FLA on interpretation learning and cognitive load. An adapted FLCAS was used to measure the specific interpretation of classroom FLA (ICFLA). Factors underlying ICFLA and the effect of ICFLA on students’ self-perceived English ability were also explored. Five research questions were posed:

1. Is ICFLA related to learners’ interpretation of learning outcomes?
2. Is ICFLA related to DD?
3. Do CI and sight translation (ST) entail different cognitive demands?
4. What are the factors underlying ICFLA?
5. Is ICFLA related to students’ language competence (self-perceived)?

## Materials and methods

### Participants

The subjects were 49 undergraduate and graduate students enrolled in English–Chinese interpretation classes at a university in Hong Kong. Twenty of them were undergraduates (14 in Year 4 and 6 in Year 3) and 29 graduate students; there were 43 females and 6 males.

The courses were elective and students were mainly trained in CI and ST. In CI training, they normally engaged in a two-stage process in which they are required to listen to the speech first, and then start translating it orally into the target language right after the speaker pauses. In ST exercises, they were instructed to process the written text in the source language and translate it orally into the target language. They had a range of years of exposure to interpretation training.

### Instruments

The questionnaire had three sections. The first was a scale to measure students’ FLA levels in their interpretation classes. The scale was designed by the first author with reference to the 33-item FLCAS by Horwitz et al. (1986). Only the items relevant to interpretation classes were included, and the wording was adapted to suit interpretation learning. For example, item 1 in the FLCAS (“I never feel quite sure of myself when I am speaking in my foreign language class”) was changed to “I never feel quite sure of myself when I am speaking English in my interpretation class.” The adapted FLCAS comprised 15 items. The FLCAS 5-point Likert rating scale was retained in the adapted one, ranging from 1 (strongly disagree) to 5 (strongly agree). Some of the items were negatively worded and reverse-scored during the analysis. The internal consistency of the scale using Cronbach’s alpha was 0.86, indicating fairly high reliability. The second section gathered students’ demographic information, including age, gender, grade level, and years of training in interpretation. The third section examined the students’ self-perceived English and interpreting competence, and the items were rated on a five-point Likert scale.

### Data collection and analysis

The questionnaires were administered to the participants in class at the end of the semester during which they had taken interpretation classes on a weekly basis. The researchers first assured the students that the data collected would be used only for research purposes and that their participation was entirely voluntary. The participants signed a consent form before filling in the questionnaire, which took approximately 15 min to complete. Learning achievement was assessed using test scores from two quizzes that covered English-to-Chinese and Chinese-to-English ST and CI. Here is the design of the quizzes: Quiz 1: ST (English to Chinese), CI (Chinese to English); Quiz 2: ST (Chinese to English), and CI (English to Chinese). The test materials were authentic speeches or materials covering various topics (e.g., ceremony, international exchange, foreign policy, science, and education). The quizzes tested the students’ ability in translating orally the source text they had read (in ST) or heard (in CI). The Chinese-to-English ST and CI parts of the tests, which are texts in English, were transcribed and used for DD analysis. There are around 569 Chinese characters on average in a CI source text and 457 Chinese characters on average in an ST source text. The data obtained from the questionnaires were analyzed using SPSS.

## Results

### Descriptive statistics

Table 1 reports descriptive statistics of the gender and grade levels of the subjects.

**TABLE 1:** Descriptive statistics of gender and grade level.

		Frequency	Percent	Cumulative percent
Gender	Male	6	12.2	12.2
	Female	43	87.8	100.0
	Total	49	100.0	
Grade level	Undergraduate students	20	40.8	40.8
	Graduate students	29	59.2	100.0
	Total	49	100.0	

Table 2 reports descriptive statistics of test scores, self-perceived language competence, and ICFLA scores.

**TABLE 2:** Descriptive statistics of test scores, SPLC, and interpretation classroom foreign language anxiety (ICFLA) scores.

	N	Minimum	Maximum	Mean	SD
Test score	49	69.25	89.75	80.45	4.68
CI test score	49	66.00	88.50	78.76	5.81
ST test score	49	67.50	91.00	81.52	5.32
SPLC	48	12.00	25.00	19.77	2.78
ICFLA	49	20.00	66.00	42.65	7.80

SPLC, self-perceived language competence.

### Foreign language anxiety and interpretation performance

Pearson product–moment correlation analysis was used to examine whether interpretation classroom foreign language anxiety (ICFLA) was correlated with student learning achievement in interpretation classes. As shown in Table 3, there was a significant negative correlation between ICFLA levels and average test scores. This result suggests that the higher the students’ language anxiety levels, the lower their test scores were likely to be.

**TABLE 3:** Pearson product–moment correlation between interpretation classroom language anxiety levels and average test score at a Hong Kong tertiary institution.

Correlation			

		Average test score	ICFLA
Average test score	Pearson correlation	1	−0.32*
	Sig. (2-tailed)		0.02
ICFLA	Pearson correlation	−0.32*	1
	Sig. (2-tailed)	0.02	

*Significant at the 0.05 level (2-tailed).

### Interpretation of classroom foreign language anxiety and test type

Although the students’ foreign language anxiety levels showed a significant negative correlation with their CI scores, as seen in Table 4, they were not significantly correlated with their ST scores. These results suggest that higher anxiety levels were related to lower consecutive test scores but not necessarily to ST scores.

**TABLE 4:** Pearson product–moment correlation between interpretation classroom foreign language anxiety levels and CI test performance.

Correlation			

		ICFLA	CI test score
ICFLA	Pearson correlation	1	−0.29*
	Sig. (2-tailed)		0.04
CI test score	Pearson correlation	−0.29*	1
	Sig. (2-tailed)	0.04	

*Significant at the 0.05 level (2-tailed).

### Interpretation of classroom foreign language anxiety and dependency distance

The audio recordings of the students’ ST and CI tests were transcribed, and the DD (indicating cognitive load) was run to calculate the students’ cognitive load while they performed ST and CI. To determine whether FLA in the interpretation classroom was related to DD, the Pearson product-moment correlation analysis between these two variables was computed. Table 5 shows a significant negative correlation between ICFLA level and DD during the CI tests. This suggests that the higher the students’ ICFLA level, the shorter the DD and the higher the cognitive load for students. However, no significant correlation was found between ICFLA levels and DD in the ST tests.

**TABLE 5:** Pearson product-moment correlation between interpretation classroom foreign language anxiety levels and dependency distance at a Hong Kong institution (CI test).

Correlation			

		ICFLA	DD in CI test
ICFLA	Pearson correlation	1	0.30*
	Sig. (2-tailed)		0.04
DD in CI test	Pearson correlation	0.30*	1
	Sig. (2-tailed)	0.04	

*Significant at the 0.05 level (2-tailed).

Factor structure of the adapted FLCAS measuring ICFLA to explore the factor structure of the 15-item adapted FLCAS, principal component analysis with varimax rotation was conducted. The selection of the best-rotated solution was based on the eigenvalues > 1 and scree test criteria. Four components had eigenvalues greater than 1 and accounted for 65.305% of the total variance.

Table 6 shows the rotated component matrix (sorted by factor). Factor 1 was defined by seven items (Q5, Q8, Q9, Q13, Q6, Q12, and Q1) mainly related to fear of speaking in class. This factor was labeled “public speaking fear.” Factor 2 was defined by three factors (Q2, Q11, and Q10) related mainly to difficulty in understanding the source text. This factor was labeled “listening comprehension difficulty.” Factor 3 was defined by three items (Q3, Q7, and Q15) mainly about fear of speaking in front of peers. This factor was labeled “fear of negative evaluation by peers.” Factor 4 comprised two items (Q4 and Q14) related to nervousness around native speakers and was labeled “apprehension about communicating with native speakers.”

**TABLE 6:** Factor analysis of ICFLCAS.

Rotated component matrix				
	
	Component			
	
	1	2	3	4
Q5. I feel confident when I speak English in interpretation classes.	**0.75**			
Q8. I feel very self-conscious about speaking English in front of other students.	**0.72**			
Q9. I get nervous and confused when speaking English in my interpretation class.	**0.68**			
Q13. I am afraid that the other students will laugh at me when I speak English.	**0.64**			
Q6. I am afraid that my interpretation teacher is ready to correct every mistake I make in English.	**0.57**			
Q12. I feel overwhelmed by the large vocabulary you have to learn to speak English.	**0.55**			
Q1. I never feel quite sure of myself when speaking English in my interpretation class.	**0.55**			
Q2. It frightens me when I don’t understand the source text in English.		**0.74**		
Q11. I feel overwhelmed by the number of rules you have to learn to speak English.		**0.69**		
Q10. I get nervous when I don’t understand every word in the English source text.		**0.68**		
Q3. I keep thinking that the other students in the interpretation class are better at language than I am.			**0.83**	
Q7. I always feel that the other students speak English better than I do.			**0.76**	
Q15. I feel embarrassed to open my mouth because I think I have poor pronunciation and intonation.			**0.57**	
Q4. I would not be nervous speaking English with native speakers.				**0.87**
Q14. I would probably feel comfortable around native speakers of English.				**0.55**

### Interpretation classroom foreign language anxiety level and self-perceived language/interpretation competence

The correlation between self-perceived language competence (five aspects) and FLA in the interpretation classroom was explored, and a Pearson product-moment correlation analysis was conducted on the relationships between the ICFLA Levels and each of the abovementioned self-perceived competencies. As displayed in Table 7, significant negative correlations were found between the students’ foreign language anxiety levels and their self-perceived competence in interpretation and foreign language (English) learning except for their self-perceived English reading and writing skills.

**TABLE 7:** Pearson product–moment correlation between interpretation classroom foreign language anxiety levels and self-perceived language competence.

	Anxiety	
	
	Pearson correlation	Sig. (2-tailed)
Self-perceived overall English ability	−0.35*	0.01
Self-perceived English speaking skill	−0.56**	0.00
Self-perceived English listening skill	−0.39**	0.01
Self-perceived English reading skill	–0.082	0.58
Self-perceived English writing skill	–0.20	0.18
Self-perceived interpreting skill	−0.62**	0.00

*Correlation is significant at the 0.05 level (2-tailed); **Correlation is significant at the 0.01 level (2-tailed).

## Discussion

Numerous studies over more than three decades have shown that FLA negatively affects students’ language learning. However, relatively few studies have explored FLA in an interpreter training context, and its effect in interpretation classes is still unknown. Some studies (e.g., Chiang, 2009, 2010) have identified negative correlations between FLA and interpretation achievements, but the conceptualization and instruments used were not directly related to FLA in interpretation classes. For example, Chiang (2009, 2010) used the 33-item FLCAS (Horwitz et al., 1986) to measure students’ FLA levels. Many FLCAS items are relevant only for foreign language classes and not for interpretation classes. For example, item 26 in the FLCAS includes the statement: “I feel more tense and nervous in my English class than in my other classes.” In the present study, the undergraduate and graduate student participants no longer had English classes in their curriculum. Therefore, if comparable items had been used, the participants would have had to recall situations from secondary school when they last took English classes. If the “English class” is changed to “interpretation class,” this item would not have directly addressed their FLA experiences during interpreting classes, because students may feel tense or nervous for other reasons in interpretation classes, for example, their poor translation skills. In contrast, the 15-item ICFLA asks students questions that are directly related to their FLA experience in interpretation classes, which enabled this study to identify the degrees and impact of FLA during interpretation training. Indeed, the appropriate design and use of an instrument to measure FLA in interpretation classes are important in foreign language classes, a setting in which the FLCAS has contributed greatly to the understanding of the roles of FLA.

As expected, a clear-cut negative correlation was identified between FLA and interpretation learning when the appropriate instrument was used. In the two classes examined in this study, two types of skill were tested: CI and ST. In CI, the students listened to the source text and then interpreted it. In ST, the students read the source text. The ICFLA scores correlated with the CI scores but not with the ST scores. This implies that the students felt more anxious during CI and found the task more difficult when listening to the source text than when reading it.

Interestingly, the correlation between ICFLA and DD in the CI test also echoes the above findings. That is, language anxiety was correlated with CI but not with ST. This finding may imply that CI is cognitively more demanding than ST. Liang et al. (2017) also found that different types of interpretation yielded different DDs; specifically, CI entailed the smallest DD and imposed heavier cognitive demands than simultaneous interpretation. This finding has been corroborated by a series of interpreting studies from multiple different linguistic perspectives, such as lexical simplification, lexical category distribution, language sequences, and syntactic networks (Liang et al., 2019; Lv and Liang, 2019; Jia and Liang, 2020; Lin et al., 2021).”

Compared with CI, ST is considered to be closer in nature to simultaneous interpreting, and it is often used as a preparatory exercise for simultaneous interpreting practices. In this sense, this study’s results confirmed the finding of Liang et al. (2017) that CI is cognitively more demanding than simultaneous interpreting. This study is the first to examine the relationship between FLA and DD in classroom learning. More investigations along this line could yield further insights into how FLA affects students’ language and interpretation learning.

Four factors were identified underpinning ICFLA: “fear of public speaking,” “difficulty in listening comprehension,” “fear of negative evaluation by peers,” and “apprehension about communicating with native speakers.” During interpretation classes, students are required to listen to (as source text) and speak (translate orally as target text or answer the instructor’s questions) in a foreign language that they are still learning. This explains the identification of the first two factors, which are related to speaking and listening. In addition, during CI training, students are likely to be required to speak in front of others, while simultaneous interpretation is conducted in booths. Therefore, the first factor is intuitively related to CI. The third factor, “fear of negative evaluation from peers,” is easily understood because students are frequently required to perform in front of their peers, whose language and interpreting skills vary greatly. The fourth factor, which relates to communicating with native speakers, is also explicable because interpretation activities usually serve native foreign language speakers’ needs. However, students often report that the natural speech of native foreign language speakers is difficult to follow because of their fast speed, wide-ranging vocabulary, and complex grammatical structures. The four factors underpinning ICFLA are related to, but different from the three components of FLA, which are communication apprehension, test anxiety, and fear of negative evaluation in the foreign language classroom (Horwitz et al., 1986), indicating unique features of their respective learning context.

Students’ self-perceptions of their learning achievements have been found to be highly reliable, which is helpful when estimating their real level of achievement (Cheng, 2002; Yan and Horwitz, 2008; Yan et al., 2010; Yan and Wang, 2012). Using the ICFLAS, the students’ FLA levels were found to be significantly negatively correlated with their self-perceived overall English ability and listening and speaking skills, but not with their self-perceived reading and writing skills. These results are reasonable because interpreting activities involve only listening and speaking. In addition, the higher the students’ FLA levels, the lower their perceived listening and speaking skills. FLA had a greater effect on the students’ self-perceived speaking skills than on any other measured item, as shown by this relationship having the largest correlation coefficient. FLA was also significantly negatively correlated with the students’ self-perceived interpretation skills, with a correlation coefficient of −0.615; this was much larger than the correlation coefficient between FLA and the students’ real interpretation achievement (−0.293). Therefore, it is likely that FLA affected the students’ self-perceptions and real performance in turn.

## Conclusion

Adapting the FLCAS and using it to measure specifically FLA in interpretation classrooms, this study found clear-cut negative correlations between FLA and students’ CI achievements, self-perceived interpretation ability, and speaking and listening skills. These findings indicated the negative effects of FLA in interpretation classes. More importantly, this study revealed a negative relationship between FLA and DD in CI, which showed that the more anxious the students were, the heavier their cognitive burden during CI. However, in contrast to CI, ST was not significantly affected by FLA and the students were less cognitively burdened by ST activities. Four factors were found to underlie the construct of ICFLA: “fear of public speaking,” “difficulty in listening comprehension,” “fear of negative evaluation by peers,” and “apprehension about communicating with native speakers.” These findings provide useful insights for researchers and educators to understand the nature of FLA in different settings and facilitate appropriate methods for reducing its effect.

The findings have important implications for classroom teaching. In interpretation classes, there are different types of anxiety. The findings of this study may help teachers differentiate FLA from other types of anxiety. Arrangements can be made to facilitate students coping with FLA. For example, teachers may discuss with the students the different speaking styles of native and non-native speakers; let students talk with their peers before inviting an individual student to answer the teacher’s questions or demonstrate interpretation in class; adjust the speaking speed of the speakers so that the students can gradually improve their listening comprehension in a foreign language; help students conduct guided peer evaluation.

Although the present study has revealed some interesting and important findings in FLA and interpretation learning, several limitations can be found. First, the problem is the small sample size, which resulted from the small interpretation classes. A larger sample size may reveal more interesting and convincing findings. Second, some important relationships have been established between several variables. More investigations on various factors associated with the relationship are needed in the future. In addition, qualitative investigations like focus groups or individual interviews can be conducted to find out more about students’ FLA and cognitive load in interpretation classes.

## Data availability statement

The raw data supporting the conclusions of this article will be made available by the authors, without undue reservation.

## Ethics statement

The studies involving human participants were reviewed and approved by Human Subjects Ethics Sub-Committee, City University of Hong Kong. The patients/participants provided their written informed consent to participate in this study.

## Author contributions

JXY conceived and designed the research, collected the data, analyzed the data except for the part related to dependency distance, and wrote the manuscript. JL performed the data analysis concerning dependency distance. Both authors contributed to the article and approved the submitted version.
